# Correlating programmed death ligand 1 (PD-L1) expression, mismatch repair deficiency, and outcomes across tumor types: implications for immunotherapy

**DOI:** 10.18632/oncotarget.20492

**Published:** 2017-08-24

**Authors:** Seung Tae Kim, Samuel J. Klempner, Se Hoon Park, Joon Oh Park, Young Suk Park, Ho Yeong Lim, Won Ki Kang, Kyoung-Mee Kim, Jeeyun Lee

**Affiliations:** ^1^ Division of Hematology-Oncology, Department of Medicine, Samsung Medical Center, Sungkyunkwan University School of Medicine, Seoul, Korea; ^2^ The Angeles Clinic and Research Institute, Los Angeles, CA, USA; ^3^ Samuel Oschin Comprehensive Cancer Institute, Cedars-Sinai Medical Center, Los Angeles, CA, USA; ^4^ Department of Pathology & Translational Genomics, Samsung Medical Center, Sungkyunkwan University School of Medicine, Seoul, Korea

**Keywords:** programmed death-ligand 1 (PD-L1), mismatch repair deficiency (dMMR), immunotherapy, biomarker, gastrointestinal cancer

## Abstract

The identification of biomarkers associated with response to therapeutic agents is central to optimizing patient outcomes. Expression of the immune checkpoint proteins PD-1/L1, and DNA mismatch repair deficiency (dMMR) status may be predictive response biomarkers for immunotherapies, but their overlap requires further study. We prospectively conducted PD-L1 and MMR immunohistochemistry (IHC) on 430 consecutive patients with advanced gastrointestinal (GI) cancers, genitourinary (GU) cancers or rare cancers between June 2012 and March 2016. Overall 393/430 (91.4%) patients were evaluable for PD-L1 expression by IHC. The frequency of tumor PD-L1 positivity (PD-L1+) was 16.5% (65/393). Among anatomic tumor sites PD-L1+ was 28.6% in melanoma, 22.2% in GC, 20.9% in CRC, 12.5% in BTC, 7.1% in GU cancer, 6.7% in HCC, 0% in pancreatic cancer and 0% in sarcoma. Among the 394 evaluable for MLH1/MSH2 expression cases, 18 patients (4.5%) had dMMR tumors. The dMMR was most common in GC (7.1%) followed by 6.7% in HCC, 4.4% in CRC, and 2.7% in sarcoma. Of the 365 patients evaluable for both PD-L1 and MLH1/MSH2 expression, there was a significant association between the PD-L1 expression and MLH1/MSH2 loss (*P* = 0.01), but not with overall survival within tumor types. PD-L1 status and dMMR are overlapping putative response biomarkers in immunoncology. Clinical trials with biomarker enrichment restricted to PD-L1+ or dMMR may be inadequate to capture the subset of patients who may benefit from immune mediated therapies. More robust immunotherapy biomarkers and careful clinical trial design are warranted.

## INTRODUCTION

Immune evasion is a hallmark of cancer and the discovery and therapeutic targeting of immune checkpoints has redefined the treatment of multiple tumors types [1]. Broadly, immune checkpoints are characterized by stimulatory or inhibitory functions and overexpression of inhibitory checkpoints by tumor or immune cells can dampen autoimmunity, form an immunosuppressive microenvironment and drive immune tolerance and escape [2, 3]. Targeting either the inhibitory checkpoint programmed cell death protein-1 (PD-1) or its ligand (PD-L1) with inhibitory monoclonal antibodies has restored antitumor immunity across multiple tumor types [4–8]. Not surprisingly, immunohistochemical (IHC) tumor and/or immune cell expression of PD-1 and/or PD-L1 has been associated with numerically higher response rates in checkpoint inhibitor trials [6, 9, 10]. However, responses are observed independent of PD-1/L1 status and it is now well described that higher tumor neoantigen burden identifies more immunogenic tumors and is associated with increased responsiveness [11].

The mismatch repair (MMR) system is of pivotal importance for the rectification of DNA sequence mismatches during DNA replication, and loss of function of one of the MMR proteins (MLH1, MSH2, MSH6, PMS2) leads to high rates of mutations that accumulate in repetitive nucleotide regions (microsatellites). Microsatellite instability (MSI), also termed MMR deficiency (dMMR), may have an oncogenic potential when it occurs in coding regions of genes involved critical cellular function [12]. The majority of sporadic MSI tumors are caused by an epigenetic inactivation of MLH1 or MSH2 [13, 14]. MMR deficient tumors have 10-100 times more somatic mutations than MMR proficient (pMMR) tumors leading to increased neoantigen burden and immunogenicity [15–19]. In fact, dMMR tumors are known to be responsive to the anti-PD-1 antibodies nivolumab and pembrolizumab [20].

To date, trials of checkpoint inhibitors have been characterized by overall response rates in the 15-25% range, but durable benefit in responding patients. Beyond PD-1/L1 IHC and MMR status, candidate predictive response biomarkers include tumor-infiltrating lymphocytes, T cell receptor (TCR) clonality, and immune gene signatures among others [21–24]. Biomarkers to more clearly define patients likely, or unlikely, to benefit is an ongoing need [25]. Importantly, the association between MMR status and PD-L1 IHC status is not well studied across different tumor types. To investigate the relationship between these biomarkers we prospectively conducted PD-L1 and MLH1/MSH2 expression in a clinically annotated cohort of patients with advanced gastrointestinal (GI) cancer, genitourinary (GU) cancer or rare cancers.

## RESULTS

### Patient characteristics

Clinicopathologic characteristics of 430 patients are listed in Table 1. The median age of the patients was 59.0 years (range, 19.0-89.0) and the male to female ratio was 1.37. The most frequent tumor type was colorectal cancer (CRC) (*n* = 203, 47.2%) followed by gastric cancer (GC) (*n* = 85, 19.8%), GU cancer (*n* = 46, 10.7%), sarcoma (*n* = 38, 8.8%), biliary tract cancer (BTC) (*n* = 16. 3.7%), hepatocellular carcinoma (HCC) (*n* = 15, 3.5%), melanoma (*n* = 8, 1.9%), pancreatic cancer (*n* = 6, 1.4%), with 13 (3.0%) other various tumor types. Over three quarters (78.6%, 338/430) of patients had stage IV disease at enrollment and 173 of 338 stage IV patients (51.2%) had 2 or more metastatic sites.

**Table 1:** Clinicopathologic characteristics of 430 patients with selected solid tumors evaluated for PD-L1 and MMR status

Clinicopathologic variable		Sample size (*n*)	Percent of total
**Gender**	Male	249	57.9%
	Female	181	42.1%
**Age**	Median (Range)	59.0 (19.0-89.0)	
	≤ 65	303	70.5%
	> 65	127	29.5%
**Tumor Type**	Gastric cancer (GC)	85	19.8%
	Colorectal cancer (CRC)	203	47.2%
	Genitourinary tract cancer	46	10.7%
	Biliary tract cancer	16	3.7%
	Pancreatic cancer	6	1.4%
	Sarcoma	38	8.8%
	Melanoma	8	1.9%
	Hepatocellular carcinoma	15	3.5%
	Miscellaneous	13	3.0%
**Disease extent**	Locally advanced disease	92	21.4%
	Metastatic disease	338	78.6%
**No. of metastatic sites**	1	165	48.8%
	≥ 2	173	51.2%

### PD-L1 expression according to tumor types

**Figure 1:**
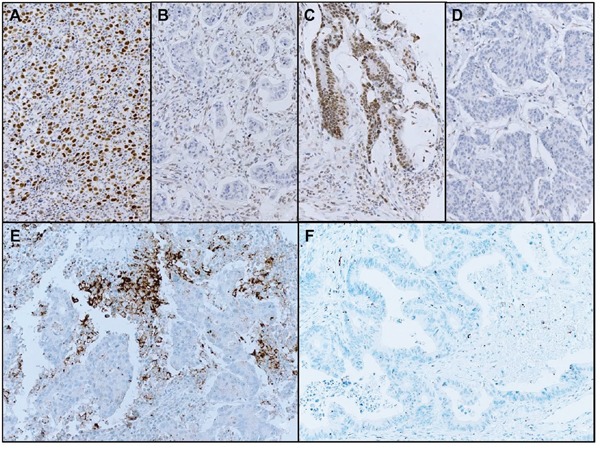
Representative histologic images of MLH1 (**A**, preserved; **B**, lost), MSH2 (**C**, preserved; **D**, lost) and PD-L1 (**E**, positive; **F**, negative) staining used in analysis of 430 patient with solid tumors.

Nearly all patients (393/430, 91.4%) were evaluable for PD-L1 expression by IHC. Among all evaluable sample 16.5% (65/393) were PD-L1+ using the ≥1% threshold defined for our study (Figure 1). Table 2 shows the status of the PD-L1 expression according to tumor types. The PD-L1 expression was positive in: 28.6% of melanoma, 22.4% of GC, 20.9% of CRC, 12.5% of BTC, 7.1% of GU cancer/miscellaneous tumors, 6.7% of HCC, 0.0% of pancreatic cancer, and 0.0% of sarcoma.

**Table 2:** PD-L1 expression by immunohistochemistry (IHC) across anatomic tumor types

Tumor type	Total (n = 430)	PD-L1+	PD-L1-	Non-evaluable
**Gastric cancer (GC)**	85	19 (22.4%)	62 (72.9%)	4 (4.7%)
**Colorectal cancer (CRC)**	203	38 (18.7%)	143 (70.4%)	22 (10.8%)
**Genitourinary cancers (GU)**	46	3 (6.5%)	39 (84.8%)	4 (8.7%)
**Biliary tract cancer (BTC)**	16	2 (12.5%)	14 (87.5%)	0 (%)
**Pancreatic cancer**	6	0 (0.0%)	6 (100.0%)	0 (0.0%)
**Sarcoma**	38	0 (0.0%)	32 (84.2%)	6 (15.8%)
**Melanoma**	8	2 (25.0%)	5 (62.5%)	1 (12.5%)
**Hepatocellular carcinoma (HCC)**	15	1 (6.7%)	14 (93.3%)	0 (0.0%)
**Other**	13	1 (7.7%)	12 (92.3%)	0 (0.0%)

PD-L1 positivity is defined as ≥1% of tumor cells staining with the SP142 PD-L1 antibody clone.

### MLH1/MSH2 expression according to tumor-types

Tumor-samples from 36 patients (8.3%) were not sufficient to analyze MLH1/MSH2 expression by IHC. Among the 394 patients available to evaluate the MLH1/MSH2 expression, 18 patients (4.4%) had MMR-deficient (dMMR) tumors with the loss of either MLH1 or MSH2 expression. Table 3 describes the status of the MLH1/MSH2 expression according to tumor-types. The MMR-deficient tumors with the loss of MLH1/MSH2 expression were observed as follows; 7.1% in GC, 6.7% in HCC, 4.4% in CRC, and 2.7% in sarcoma.

**Table 3:** MLH-1/MSH-2 expression by immunohistochemistry (IHC) according to tumor-types across a cohort of 430 patients with solid tumors

Tumor type	Total (n = 430)	MLH1/MSH2 intact (pMMR)	MLH1/MSH2 loss (dMMR)	Non-evaluable
**Gastric cancer (GC)**	85	75 (88.2%)	6 (7.1%)	4 (4.7%)
**Colorectal cancer (CRC)**	203	184 (90.6%)	9 (4.4%)	10 (5.0%)
**Genitourinary cancers (GU)**	46	36 (78.3%)	1 (2.2%)	9 (19.5%)
**Biliary tract cancer (BTC)**	16	16 (100.0%)	0 (0.0%)	0 (0.0%)
**Pancreatic cancer**	6	6 (100.0%)	0 (0.0%)	0 (0.0%)
**Sarcoma**	37	33 (89.2%)	1 (2.7%)	3 (8.1%)
**Melanoma**	8	7 (87.5%)	0 (0.0%)	1 (12.5%)
**Hepatocellular Carcinoma (HCC)**	15	8 (53.3%)	1 (6.7%)	6 (40.0%)
**Other**	14	11 (78.6%)	0 (0.0%)	3 (21.4%)

### Correlation between PD-L1 and MLH1/MSH2 expression

**Figure 2:**
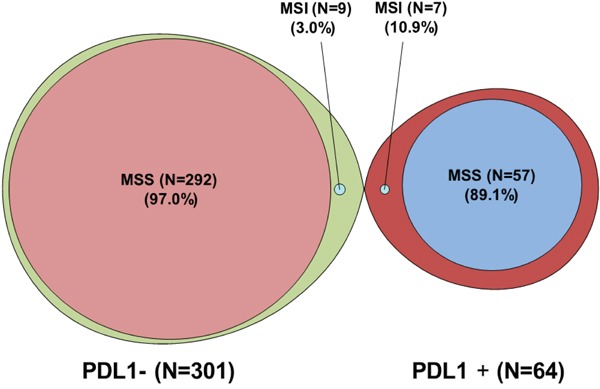
Graphical representation showing the overlap between PD-L1 status and MMR status (MLH1/MSH2) among a cohort of 365 solid tumors available for both PD-L1 status and MMR status (MLH1/MSH2) Among PD-L1+ samples there is enrichment for dMMR (MSI) when compared to PD-L1- samples.

Among the 365 patients evaluable for both PD-L1 and MLH1/MSH2 expression, we analyzed the correlation between PD-L1 and MMR status (Figure 2). The expression of PD-L1 was significantly associated with dMMR (*P* = 0.01). In the overall cohort (*n* = 430), PD-L1 expression was observed in 38.9% (7/18) of dMMR tumors (MLH1/MSH2 loss), 15.2% (57/376) of MMR proficient (pMMR) tumors, and 2.8% (1/36) tumors with unknown MMR status.

### The impact of PD-L1 expression and/or MLH1/MSH2 loss on patients’ survival

**Figure 3:**
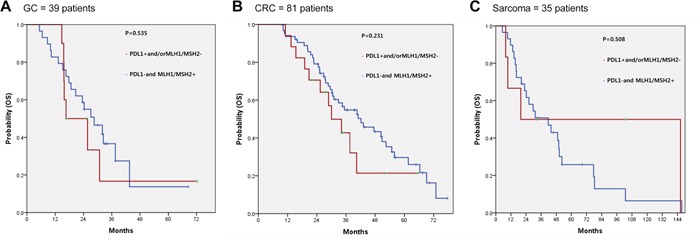
Impact of PD-L1 and MLH1/MSH2 IHC status on overall survival (OS) among cohorts of GC **(A)**, CRC **(B)**, and sarcomas **(C)** who had received all standard of care therapies.

Among patients treated with all standard therapies for their respective tumor types, the influence of PD-L1 expression and/or MLH1/MSH2 loss on survival was evaluated, stratified by anatomic tumor type. Data is available for metastatic GC, CRC, and sarcoma. Among 39 metastatic GC patients who received 2 or more lines of therapy there was no significant difference in overall survival (OS) between patients with and without PD-L1 expression and/or MLH1/MSH2 loss (*P* = 0.535) (Figure 3A). In 81 metastatic CRC patients who failed irinotecan, oxaliplatin, fluorouracil (FU) and/or bevacizumab/cetuximab containing regimens, and 35 metastatic sarcoma patients who failed doxorubicin-based therapies, PD-L1 expression and/or MLH1/MSH2 loss did not affect the OS (*P* = 0.231 and *P* = 0.508 respectively) (Figure 3B-3C)

## DISCUSSION

The identification of prognostic and predictive biomarkers in oncology is of central importance. Prospectively validated predictive response biomarkers can identify patients most likely to benefit from a given therapy while sparing potential physical and socioeconomic toxicity in those unlikely to benefit. In the rapidly evolving field of immunoncology, checkpoint overexpression (PD-1/L1) by IHC and more recently MMR status (IHC and/or PCR) are emerging biomarkers, though little is known about their relationship to each other [5–8, 20]. In the present study, we identified PD-L1 positivity (≥1% tumor cells, SP142 Ab clone) in 16.5% of samples from a large cohort of 430 clinically annotated solid tumor patients, and noted significant relationship with MMR status across anatomic tumor types (*P* = 0.01). Further, in cohorts of advanced GC, CRC, and sarcoma there was no association between PD-L1 status, MMR status, and survival.

PD-L1 expression is a dynamic process and overexpression is reliably detected by multiple IHC-validated antibodies in tumor and or immune cells [26]. To facilitate clinical relevance, we utilized the SP142 anti-PD-L1 antibody, which a US FDA approved antibody and the companion diagnostic for atezolizumab in advanced urothelial cancers [27]. Variability of PD-L1 expression is reported in different tumor types, and differing assays and thresholds have impaired cross compound comparison and universal adoption [22, 28, 29]. Intra-patient heterogeneity, the reliability of detection methods and lack of standard cut-off value for the PD-L1 expression further confound PD-1/L1 as a universal biomarker. Support for utilizing PD-L1 for patient selection comes from a recent meta-analysis including twenty trials and 1,475 patients. The meta-analysis revealed that the overall response rate of patients with PD-L1 expression is significantly higher than those without PD-L1 expression (34.1% vs. 19.9%, *P* < 0.0001) [30]. However, response rates of 10-20% in PD-1/L1 IHC negative patients confirms that further biomarker work is needed [5, 31, 32].

Tumors with germline or acquired somatic alterations in the main mismatch repair (MMR) genes (*MLH1, MSH2, MSH6, PMS2*) accumulate genomic alterations and dMMR is a proxy for elevated tumor mutational burden and presumably neoantigen load [33]. Tumors with dMMR are known to have particularly high response rates and clinical benefit from checkpoint inhibitors, most well studied in GI cancers [20, 34]. Our study results for dMMR are largely consistent with the reported frequencies of dMMR in advanced CRC (~5%) suggesting our patient population is representative [15]. Outside of patients with inherited cancer syndromes MLH1 and MSH2 assessment captures the vast majority of dMMR tumors and was utilized in our study and others. The trivial fraction of PMS2 or MSH6 loss not captured by our approach is unlikely to influence our findings [35–37].

The large majority of patients had available tissue for assessing both PD-L1 and dMMR status, and we demonstrated a correlation between these biomarkers across common tumor types. Mechanistically, the relationship between PD-L1+ and dMMR may be related to the increased neoantigen load resulting from dMMR which induces immune recognition and response, often pathologically supported by increased tumor infiltrating lymphocytes, an environment where tumor cells might be expected to increase PD-1/L1 [38]. Others have reported increased PD-L1 expression in dMMR endometrial cancers, yet not enriched in dMMR colon cancers, where only 12.5% were PD-L1+ [15–17, 39]. A recent series of CRC using the E1L3N (Cell Signaling, Danvers, MA) antibody clone to assess PD-L1 IHC suggests increased PD-L1+ in dMMR tumors with 18% dMMR CRC staining positive versus 2% of pMMR CRC [40]. Again, variability in positive/negative thresholds complicate comparison among these datasets, and highlight the need for more robust pan-cancer biomarkers. Importantly, the majority of dMMR tumors do not express PD-L1 (our data and others), suggesting that a PD-L1 restricted patient selection strategy would miss most dMMR tumors, a subgroup known to be responsive.

Our data expands on the current understanding of PD-L1 status and dMMR by examining multiple anatomic tumor types, not previously well studied. We identify PD-L1 expression in 38.9% (7/18) dMMR tumors (MLH1/MSH2 loss), 15.2% (57/376) of MMR proficient (pMMR) tumors, and 2.8% (1/36) tumors with unknown MMR status (Figure 2). Among gastric, colon, and sarcoma patients in our cohort who progressed on standard therapies there was no association between PD-L1 or MMR status with overall survival (Figure 3A-3C). Admittedly, only a small fraction (4.5%, 18/394 evaluable) of patients had dMMR tumors and the numerical increase in PD-L1+ samples did not reach statistical significance. None of the patients in our cohort received checkpoint inhibitors though we may hypothesize that the survival in dMMR tumors treated with immune therapies would be improved. Although limited by small sample size of dMMR tumors, our observed dMMR frequencies parallel what is reported for included tumor types suggesting our dataset reflects real world practice. Larger datasets from ongoing trials will be needed to refine the MMR/PD-L1 relationship.

Overall our results add to the emerging literature on immune biomarkers and support the need for improved patient selection. Numerous ongoing immunotherapy trials limit eligibility to either dMMR or PD-1/L1+ patients in a mutually exclusive manner. This approach is suboptimal and more biologically relevant biomarkers are needed. Importantly, other genomic mechanisms such as *POLE* mutations can recapitulate the elevated neoantigen burden in dMMR tumors, and are not identified by MMR or PD-1/L1 testing alone [41]. Tumor mutational burden (TMB) derived for massive parallel sequencing may emerge as a more functional biomarker in immunoncology and early data suggest superiority to PD-1/L1 IHC determination [11, 27]. Perhaps composite biomarkers such TMB-high/PD-L1+ or dMMR/PD-L1+ will identify particularly high response rates. A prospective basket trial accepting all tumors with an elevated TMB with stratification by MMR and PD-1/L1 status would be an interesting study to test optimal immune biomarkers.

## MATERIALS AND METHODS

### Patients

Patients with pathologic confirmation of advanced gastrointestinal (GI) cancer, genitourinary (GU) cancer or rare cancers at Samsung Medical Center between June 2012 and March 2016 (*n* = 430) were tested for PD-L1 and MLH1/MSH2 expression from samples taken at the time of diagnosis. All study participants provided written informed consent before study entry and were monitored in accordance with the Declaration of Helsinki. The following clinicopathologic characteristics were collected for all patients: age, gender, primary tumor site, number of metastatic sites, site of metastasis, treatment and survival.

### Tissue microarray construction and immunohistochemistry (IHC) of PD-L1

In all patients, representative tumor areas were selected and tissue microarray was constructed after review of a hematoxylin and eosin stained section from the donor block. With a guidance of this slide, two representative regions of tumor were sampled from the donor block and 2mm diameter cores were extracted and embedded in the array block. Tumor sections from array blocks were freshly cut to 4mm and dried at 60°C for 30 minutes. PDL-1 IHC (rabbit anti-human PDL-1 monoclonal, 1:25, clone SP142; Ventana, Tucson, AZ) was performed on an automated immunostainer (Benchmark; Ventana). Antigen retrieval was performed for 92 min with CC1 and the antibody was incubated for 120 min in 37°C in Ventana BenchMark XT. Signal visualization was achieved with the Optiview DAB IHC detection kit (Catalogue number 760-700) and Optiview Amplification kit (Catalogue number 860-099). PD-L1 expression was evaluated on tumor cells. The proportion of PD-L1-positive (PD-L1+) cells was estimated as a percentage of total tumor cells. Consistent with several reported clinical trials, specimens were categorized as IHC negative or positive if < 1% or ≥ 1% of cells were stained by PD-L1 mAb, respectively (Figure 1).

### Immunohistochemistry (IHC) of MLH1/MSH2

IHC was performed on tissue microarray tumor sections using antibodies against MLH1 (clone ES05; 1: 200 dilution; Leica Biosystems, Melbourne, Australia) and MSH2 (clone G219-1129; 1: 500 dilution; CELL Marque; Rocklin, CA, USA). IHC staining procedures were conducted using a Bond-max autoimmunostainer (Leica Biosystems, Melbourne, Australia) using Bond™ Polymer refine detection, DS9800 (Vision Biosystems, Melbourne, Australia). MLH1 and MSH2 proteins were determined to be preserved in a case when nuclear staining in the tumor cells of the case was observed. Loss of expression of MMR proteins (MLH1- or MSH2-) was determined when nuclear staining in the tumor cells was not observed. In the present study, MMR-deficient or MSI tumors was defined as the loss of MLH1 and/or MSH2 expression.

### Statistical analyses

Descriptive statistics were reported as proportions and medians. Data were also presented as number (%) for categorical variables. Correlation between the status of PD-L1 and MLH1/MSH2 expression was analyzed using the t-test or the Fisher's exact test as appropriate, or one-way analysis of variance (ANOVA). Overall survival (OS) was defined as the time from the first treatment to the date of death. Kaplan-Meier estimates were used in the analysis of all time to event variables, and the 95% confidence interval (CI) for the median time to event was computed.

### Ethics statement

The institutional review board of the Samsung Medical Center (SMC) approved the study. The methods in this study were carried out in accordance with the approved guidelines by SMC and all protocols were approved by the ethics committees of SMC.
